# Concentration-Invariant Odor Representation in the Olfactory System by Presynaptic Inhibition

**DOI:** 10.1155/2013/507143

**Published:** 2013-02-26

**Authors:** Danke Zhang, Yuanqing Li, Si Wu

**Affiliations:** ^1^School of Automation Science and Engineering, South China University of Technology, Guangzhou 510641, China; ^2^State Key Laboratory of Cognitive Neuroscience and Learning, Beijing Normal University, Beijing 100875, China

## Abstract

The present study investigates a network model for implementing concentration-invariant representation for odors in the olfactory system. The network consists of olfactory receptor neurons, projection neurons, and inhibitory local neurons. Receptor neurons send excitatory inputs to projection neurons, which are modulated by the inhibitory inputs from local neurons. The modulation occurs at the presynaptic site from a receptor neuron to a projection one, leading to the operation of divisive normalization. The responses of local interneurons are determined by the total activities of olfactory receptor neurons. We find that with a proper parameter condition, the responses of projection neurons become effectively independent of the odor concentration. Simulation results confirm our theoretical analysis.

## 1. Introduction

 An external physical or chemical stimulus contains multiple aspects of information, for instance, a natural image may contain the luminance, the color, and the shape information of an object, and an odorant input may contain the identity and the intensity information of an odor. These multiple aspects of information are represented by spiking activities of neuron ensembles layer by layer. It has been found that neurons often show invariant responses to certain features of stimulus, for instance, ganglion cells in the retina display some degree of spatial luminance invariance to light signals [1], and complex cells in the primary visual cortex are insensitive to the phase of orientation [2]. The invariance representation for stimulus feature is beneficial to neural information processing, as it makes a neural system concentrate on representing or extracting a special aspect of external information. It has been reported that some Kenyon cells in the locust mushroom body exhibit concentration-invariance responses to odor stimulus [3], but how does this concentration-invariance arise remains largely unknown.

For the drosophila olfactory system, odorant information is first represented by the activities of olfactory receptor neurons (ORNs) [4]. There are about 60 classes of ORNs in drosophila. The firing rates of ORNs increase with the odor concentration. ORNs expressing the same receptor gene project their axons into the same glomerulus, where they make excitatory synaptic connections with the projection neurons (PNs) in the antennal lobe. Generally, a PN innervates a single glomerulus and hence the ORN-PN connection constitutes a direct signal transmission pathway. The ORN-PN synapse is strong and exhibits short-term depression [5]; that is, the synaptic efficacy depends on the spiking history of the presynaptic neuron. It has been reported that the synapse from ORN to PN is modulated by the inhibitory inputs from local neurons [6], and the activity of the latter is determined by the total activity of ORNs. The presynaptic inhibition is known to have the effect of normalizing neuronal responses divisively. Thus, the activity of a PN is, on one hand, driven by its presynaptic ORNs, and on the other hand, modulated by the total activities of all ORNs through the presynaptic inhibition. In this study, we show that through this presynaptic inhibition, the stationary state of PNs can achieve concentration-invariant representation for an odor.

## 2. The Model

**Figure 1:**
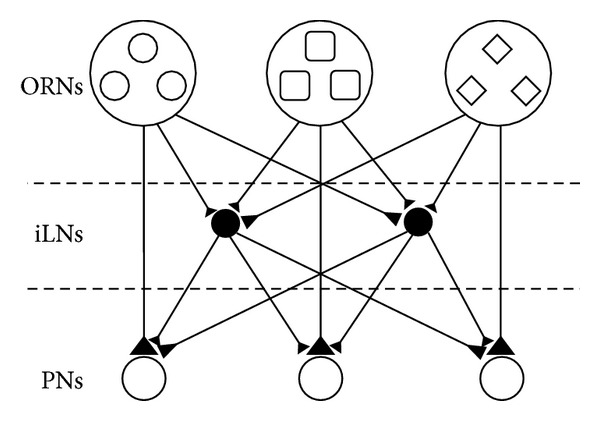
The network structure.

 We consider there are *N* ^*E*^ clusters of ORNs, each of them corresponding to one receptor expression type, and there are *K* cells in each cluster. A cluster of ORNs is connected to a PN (there are therefore *N* ^*E*^ number of PNs), and it is also connected to all inhibitory local neurons (iLNs). The synapse from an ORN to a PN holds short-term depression. The connection from an iLN to a PN locates at the terminal of the presynaptic site from an ORN to the PN; therefore, it modulates the input to the PN. There are totally *N* ^*I*^ iLN, and each of them receives inputs from all ORNs. The network structure is shown in Figure 1.

The dynamics of a single neuron is described by an integrate-and-fire process. When the membrane potential of a neuron is above a threshold, an action potential is generated and after that the membrane potential is reset to a resting value. Below the threshold, the dynamics of a neuron is given by

(1) $$\begin{array}{c} C^{T}\frac{dv_{i}^{T}}{dt}=-g_{L}\left(v_{i}^{T}-E_{L}\right)-I_{i}^{\text{syn}}, \end{array}$$

where *C* ^*T*^ is the membrane capacitance of a *T*-type neuron, with *T* = *E* or *I* denoting the neuron being excitatory or inhibitory, respectively. *g*_*L*_ is the leaky conductance, and *E*_*L*_ is the corresponding reversal potential. The synaptic current *I*_*i*_^syn^ is written as follows:

(2) $$\begin{array}{c} I_{i}^{\text{syn}}=\sum_{j}g_{ij}^{TE}\left(v_{i}^{T}-E_{E}\right), \end{array}$$

where *g*_*ij*_^*TE*^ is the excitatory conductance and *E*_*E*_ is the reversal potential.

For an inhibitory neuron, the dynamics of the conductance *g*_*ij*_^*IE*^ is given by

(3) $$\begin{array}{c} \frac{dg_{ij}^{IE}}{dt}=-\frac{g_{ij}^{IE}}{\tau_{E}}+w^{IE}\sum_{k}\delta \left(t-t_{jk}\right), \end{array}$$

where *w* ^*IE*^ is the connection strength, and *τ*_*E*_ the synaptic time constant. *g*_*ij*_^*IE*^ is driven by the activity of the *j*th presynaptic ORN. An ORN generates action potentials according to Poisson process, with *t*_*jk*_ denoting the moment of the *k*th spike of the *j*th ORN.

The synapse conductance from the *j*th ORN to the *i*th PN, *g*_*ij*_^*EE*^, exhibits short-term depression [7] and is modulated by the activities of iLNs, whose dynamics is given by

(4) $$\begin{array}{c} \frac{dg_{ij}^{EE}}{dt}=-\frac{g_{ij}^{EE}}{\tau_{E}}+w^{EE}x_{ij}f\left(g_{i}^{I}\right)\sum_{l}\delta \left(t-t_{jl}\right),\\ \frac{dx_{ij}}{dt}=\frac{1-x_{ij}}{\tau_{D}}-x_{ij}f\left(g_{i}^{I}\right)\sum_{l}\delta \left(t-t_{jl}\right),\\ \frac{dg_{i}^{I}}{dt}=-\frac{g_{i}^{I}}{\tau_{I}}+w^{I}\sum_{k}^{N^{I}}\;\sum_{m}\delta \left(t-t_{km}\right), \end{array}$$

where *x*_*ij*_ denotes the available vesicle resource in the presynaptic site of the *j*th ORN, *f*(*g*_*i*_^*I*^) is the vesicle release probability modulated by the presynaptic input, *w* ^*EE*^ is the synaptic connection strength from ORN to PN, *w* ^*I*^ is the synaptic connection strength from iLN to PN, and *τ*_*D*_ is the recovery time constant of the vesicle release. When a spike is emitted from an ORN, the synaptic conductance *g* ^*EE*^ is increased by *w* ^*EE*^ *x*_*ij*_ *f*(*g*_*i*_^*I*^) due to the opening of ion channels, and the vesicle resource in the presynaptic site is decreased by *x*_*ij*_ *f*(*g*_*i*_^*I*^). The increased conductance and decreased vesicle resource depend on the spiking history of its presynaptic ORN and the strength of presynaptic inhibition.

In the ORN-PN pathway, a lateral presynaptic inhibition is found to modulate the strength of synaptic transmission. This form of presynaptic inhibition is also found in the retina [8]. Each spike could depolarize the membrane potential in the presynaptic terminal, and the amount of membrane depolarization is modulated by the amount of inhibitory synaptic conductance. To model this effect, we assume that the vesicle release probability *f*(*g*_*i*_^*I*^) is a monotonically decreasing function of the inhibitory synaptic conductance, that is,

(5) $$\begin{array}{c} \;f\left(g_{i}^{I}\right)=\frac{1}{ag_{i}^{I}+b}, \end{array}$$

where *a* and *b* are constants.

Let us give a short summary of the synaptic transmission from ORN to PN. The synaptic connection strength from ORN to PN depends on three factors: the vesicle resource in the presynaptic site, the release probability, and the number of release sites. In the above, we model the vesicle resource with a variable *x*. The release probability is determined by the calcium concentration in the presynaptic terminal, and the latter is affected by the depolarization level of the local membrane. The presynaptic inhibitory input influences the membrane depolarization level. Thus, we model the presynaptic inhibition effect by assuming that the release probability decreases with the inhibitory input. The increment of the synaptic conductance upon a spike is modeled as a product of the available vesicle resource, the release probability, and the synaptic connection strength.

## 3. Input Gain Control in the Response of PNs

 Input gain control phenomenon is widely observed in the early pathway of a sensory system [9–12]. It is defined as that the neuronal transfer function is modulated by the magnitude of inputs. In the olfactory system, the input gain control has been observed in the ORN-PN pathway, in which the response of a PN to its presynaptic ORN inputs is modulated by total activities of ORNs [11]. In the experiment, a few odors were identified that each odor only activates a single type of ORNs. These odors are called private odors for simplicity. By varying the concentration of a private odor, the mean firing rates of ORNs and its cognate PNs are recorded. The input-output relationship shows a sigmoid-like curve function. Interestingly, it is found that the input-output function could be input gain modulated by superimposing a public odor that could activate many types of ORNs but not the ORNs activated by private odors. Here, we show that short-term depression and the presynaptic inhibition can achieve input gain control in the olfactory system.

By setting the left-hand of (3)–(4) to be zero and considering the continuum limit, we get the stationary state of the network, which is given by

(6) $$\begin{array}{c} \;g^{IE}=Kw^{IE}\sum_{i=1}^{N^{E}}r_{i}^{O}\tau_{E},\\ g_{i}^{EE}=K\frac{w^{EE}f\left(g_{i}^{I}\right)r_{i}^{O}\tau_{E}}{1+f\left(g_{i}^{I}\right)r_{i}^{O}\tau_{D}},\\ g_{i}^{I}=N^{I}w^{I}r^{I}\tau_{I}, \end{array}$$

where *r*_*i*_^*O*^ is the firing rate of the the ORNs in the *i*th cluster, and *r* ^*I*^ is the firing rate of iLNs. *g* ^*IE*^ is the total excitatory synaptic conductance received by an inhibitory neuron, *g*_*i*_^*EE*^ is the total excitatory synaptic conductance received by the *i*th PN, and *g*_*i*_^*I*^ is the inhibitory conductance at the presynaptic terminal of the ORN-PN synapse. For simplicity, we assume that the firing rate is a linear function to the synaptic conductance, that is,

(7) $$\begin{array}{c} r^{T}=\alpha^{T}\left(g^{TE}-\beta \right). \end{array}$$

Thus, we have

(8) $$\begin{array}{c} \;r^{I}=\alpha^{I}\left(Kw^{IE}\sum_{i}r_{i}^{O}\tau_{E}-\beta \right),\\ g_{i}^{I}=N^{I}w^{I}\tau_{I}\alpha^{I}\left(Kw^{IE}\sum_{i}r_{i}^{O}\tau_{E}-\beta \right). \end{array}$$

By using (5), we get

(9) $$\begin{array}{c} g_{i}^{EE}=\frac{Kw^{EE}\tau_{E}r_{i}^{O}}{aN^{I}w^{I}\tau_{I}\alpha^{I}Kw^{IE}\sum_{i}r_{i}^{O}\tau_{E}-aN^{I}w^{I}\tau_{I}\alpha^{I}\beta +b+\tau_{D}r_{i}^{O}}, \end{array}$$

and the firing rate of the *i*th PN is

(10) $$\begin{array}{c} r_{i}^{E}=\alpha^{E}\left(g_{i}^{EE}-\beta \right). \end{array}$$

**Figure 2:**
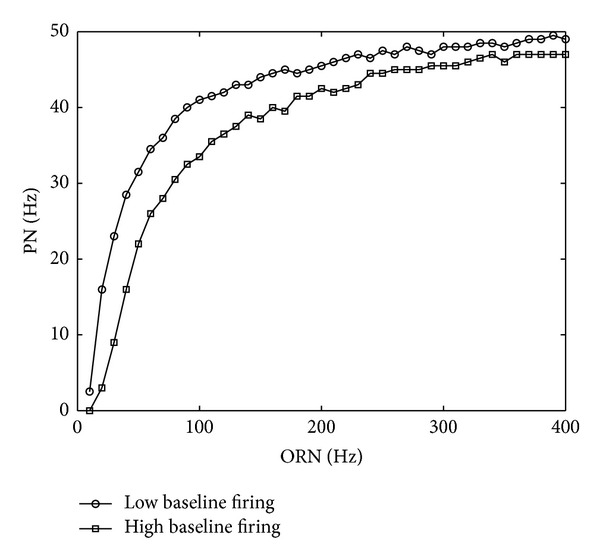
The ORN-PN response curve is modulated by the total activity of ORNs. In the low and high baseline cases, the firing rates of other ORNs are set to be 20 Hz and 30 Hz, respectively. Symbols denote the simulation results. The parameters are *N* ^*E*^ = 49; *K* = 40; *N* ^*I*^ = 20; *w* ^*EE*^ = 10 nS; *w* ^*EI*^ = 0.05 nS; *w*_*i*_^*I*^ = 0.1 nS; *τ*_*D*_ = 100 ms; *τ*_*E*_ = 5 ms; *τ*_*I*_ = 100 ms; *C* ^*E*^ = 800 pF; *C* ^*I*^ = 800 pF; *g*_*L*_ = 25 nS; *E*_*L*_ = −70 mV; *E*_*E*_ = 0 mV; *v*_th_ = −50 mV; *v*_reset_ = −70 mV; *a* = 0.3; *α* ^*I*^ = 3.76; *β* = 5.65; *b* = *aN* ^*I*^ *α* ^*I*^ *βτ*_*I*_ *w* ^*I*^.

From (9)-(10), we see that the response of a PN is modulated by the total activity of ORNs (see the denominator of (9)). Its firing rate does not increase linearly with the input strength, but saturates when the input is strong enough.  Figure 2 shows the response curves of PNs to the presynaptic ORNs under different levels of background ORN activities.

## 4. Concentration-Invariant Representation for Odors

The activity of a PN depends on two factors: the excitatory current from its presynaptic ORNs and the vesicle release probability controlled by the presynaptic input from iLNs, and the latter decreases with the total activity of ORNs. Consider an odor activates a subset of ORNs, with their firing rates *r*_*i*_^*O*^ increase linearly with the odor concentration. We see that the concentration-invariant representation holds if the following condition is satisfied, which is

(11) $$\begin{array}{c} aN^{I}w^{I}\tau_{I}\alpha^{I}\beta -b=0. \end{array}$$

With the above condition, the stationary value of excitatory conductance to a PN is written as follows:

(12) $$\begin{array}{c} g_{i}^{EE}=\frac{Kw^{EE}\tau_{E}r_{i}^{O}}{aN^{I}w^{I}\tau_{I}\alpha^{I}Kw^{IE}\sum_{i}r_{i}^{O}\tau_{E}+\tau_{D}r_{i}^{O}}. \end{array}$$

**Figure 3:**
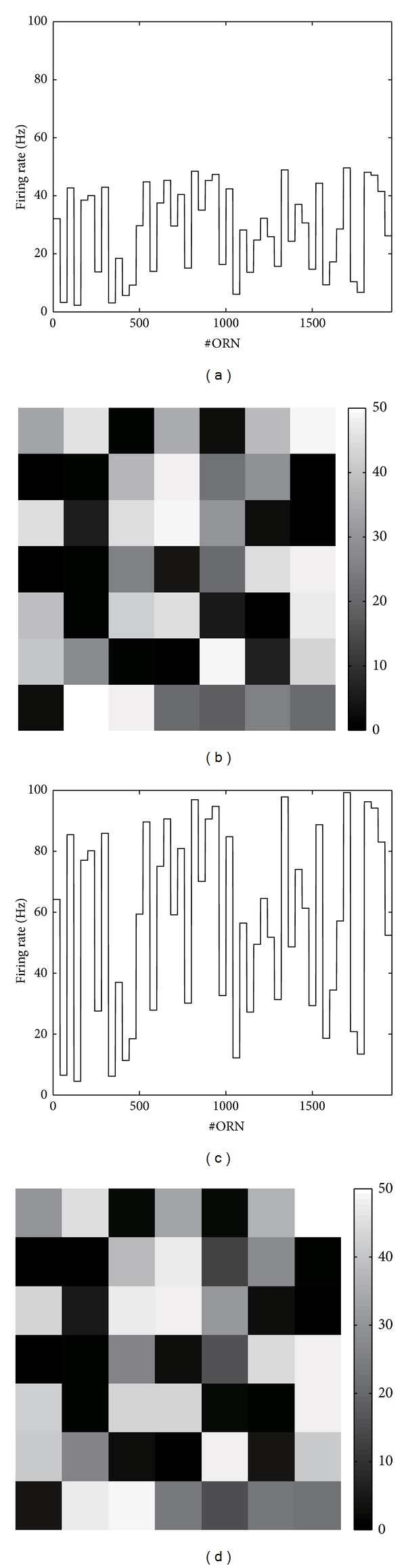
The concentration-invariant representation for an odor. The left panel shows the ORN population responses at the low and high odorant concentrations; the right panel shows the corresponding PN population responses. The population responses of PNs do not vary much in different odor concentration conditions.

We see that the linear dependence of *r*_*i*_^*O*^ on the odor concentration in the nominator and denominator of the above equation cancel each other, and hence *g*_*i*_^*EE*^, and so does *r*_*i*_^*E*^, become invariant with respect to the odor concentration.  Figure 3 shows the simulation result, which confirms the theoretical analysis.

**Figure 4:**
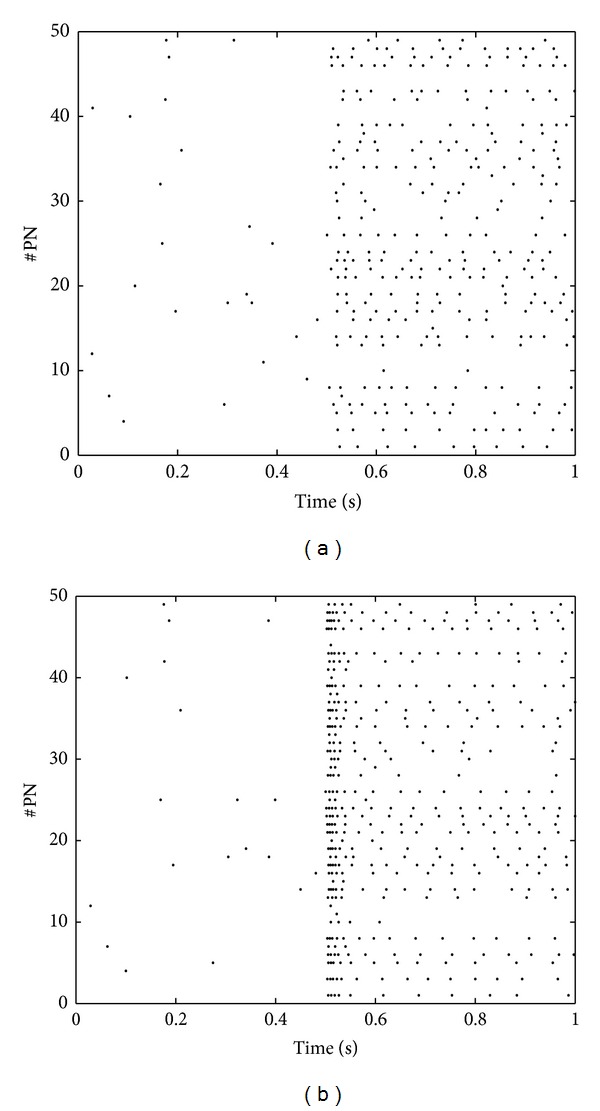
The transient dynamics of PNs in different odor concentrations. The firing rates of ORNs are uniformly distributed in the range of (0,20) Hz in the low concentration case and in the range of (0,100) Hz in the high concentration case. PN fires spontaneously before the application of the odor.

In the above, we show that the responses of PNs in stationary state only encode the identity information of an odor. The concentration information of an odor is conveyed by the transient dynamics of PNs.  Figure 4 shows the population activities of PNs shortly after the application of an odor, which demonstrates that the firing rates of PNs in the high concentration case are much higher than that in the low concentration case.

## 5. Conclusions and Discussions

 In this study, we have proposed a network model for implementing concentration-invariant representation for odors in the olfactory system. Two elements are involved in achieving this goal, namely, short-term synaptic depression and presynaptic inhibition. The former ensures the responses of PNs saturate for large inputs, and the latter modulates the excitatory inputs to PNs according to the total activities of ORNs. The modeling result is consistent with experimental findings. As gain control effect has been widely observed in neural systems, the underlying mechanisms have long been debated [13–15]. Here, we propose an input gain control mechanism mediated by presynaptic inhibition. Furthermore, we find a parameter condition under which the stationary responses of PNs become invariant with respect to the concentration of odors. It has been pointed out that input gain control can generate invariance representation [16]. Here, we show that this could be realized by presynaptic inhibition. It has been observed in experiment that Kenyon cells in mushroom body could exhibit concentration invariant responses in a certain range of odor concentrations [3]. In this study, we show that this concentration-invariant representation is achieved in the layer of PNs, which are presynaptic to Kenyon cells. The concentration information of an odor is, on the other hand, represented by the transient dynamics of PNs. It is our future work to explore the circuit temporal dynamics in detecting odor concentrations.
